# Augmentation of IFN-γ by bone marrow derived immune cells in the presence of severe suppression of IFN-γ in gingivae induced by zoledronic acid and denosumab in Hu-BLT mice model of ONJ

**DOI:** 10.3389/fendo.2023.1111627

**Published:** 2023-01-20

**Authors:** Kawaljit Kaur, Yujie Sun, Keiichi Kanayama, Kenzo Morinaga, Akishige Hokugo, Ichiro Nishimura, Anahid Jewett

**Affiliations:** ^1^ Weintraub Center for Reconstructive Biotechnology, UCLA School of Dentistry, Los Angeles, CA, United States; ^2^ Division of Oral Biology and Medicine, University of California School of Dentistry, Los Angeles, CA, United States; ^3^ Division of Advanced Prosthodontics, University of California School of Dentistry, Los Angeles, CA, United States; ^4^ Department of Periodontology, Asahi University School of Dentistry, Gifu, Japan; ^5^ Department of Oral Rehabilitation, Fukuoka Dental College, Fukuoka, Japan; ^6^ Division of Plastic Surgery, David Geffen School of Medicine at UCLA, Los Angeles, CA, United States

**Keywords:** zoledronic acid, denosumab, osteonecrosis of the jaw, NK cells, antibody-dependent cellular cytotoxicity (ADCC), humanized-BLT mice, cytotoxicity, IFN-γ

## Abstract

**Introduction:**

The potential mechanisms governing drug induced osteonecrosis of the jaw (ONJ) is not well understood, and is one of the objectives of this study. Thus, we tested the release of IFN-γ within different immune compartments including bone marrow and gingivae upon treatment with zoledronic acid (ZOL) and denosumab which are known to induce ONJ in susceptible individuals.

**Methods:**

We used humanized-BLT mouse model for the *in-vivo* studies reported in this paper. To determine the effects of zoledronic acid and denosumab on IFN-γ secretion and NK cell-mediated cytotoxicity; peripheral blood, bone marrow, spleen and gingiva were obtained after the injection of ZOL and denosumab in mice.

**Results:**

Percentages of B cells are much higher in wild-type mice whereas the proportions of immune subsets in humans and reconstituted hu-BLT peripheral-blood are similar. Therefore, hu-BLT mice are preferable model to study human disease, in particular, immune-pathologies induced by ZOL and denosumab. Both agents resulted in a severe suppression of IFN-γ in the gingiva, whereas they heightened the release of IFN-γ and NK cell-mediated cytotoxicity by the BM-derived immune cells. ZOL increased the IFN-γ secretion by the spleen and peripheral blood immune cells, whereas denosumab decreased the release IFN-γ by these cells significantly.

**Discussion:**

ZOL and denosumab may likely suppress IFN-γ secretion in gingiva through different mechanisms. In addition, to the suppression of IFN-γ secretion, denosumab mediated effect could in part be due to the decrease in the bone resorptive function of osteoclasts due to the induction of antibody dependent cellular cytotoxicity and lysis of osteoclasts, whereas ZOL is able to mediate cell death of osteoclasts directly. Suppression of IFN-gamma in gingiva is largely responsible for the inhibition of immune cell function, leading to dysregulated osteoblastic and osteoclastic activities. Restoration of IFN-gamma in the local microenvironment may result in establishment of homeostatic balance in the gingiva and prevention of osteonecrosis of jaw.

## Introduction

Bisphosphonates (BPs) are the prototypic anti-resorptive medication (1). Osteonecrosis of the jaw (ONJ), also known as BP-related ONJ (BRONJ) occurs in patients treated with BPs, mostly in the segment of jawbone interfacing oral mucosa (2). The overall prevalence of BRONJ is relatively low and an overwhelming proportion of the reported cases are related to high-dose intravenous BPs in cancer patients with multiple myeloma (55.9%) and breast cancer (33.4%) (3–5). BRONJ was first described in early 2000’s (6–8); however, the pathological mechanism and therapeutic modalities have not yet been established. Tooth extraction (9, 10), periodontitis (11) and, ill-fitting dentures (12) were reported to increase the risk of ONJ.

Oral mucosa and gingiva, which overlays the alveolar bone of the maxilla and mandible, are considered to be a functional barrier tissue with barrier immunity (13). Uniquely, the oral barrier tissue interfaces the external surface of alveolar bone. Unlike other barrier tissues, the activated immune system in the oral barrier may have a profound effect on the underlying alveolar bone (14–16). These oral risk factors are known to induce oral barrier inflammation and osteoclastogenesis. Therefore, we have hypothesized that osteoclasts associated with the oral barrier tissue may modulate and potentially define the oral barrier immune reaction under the influence of BPs.

Osteoclasts (OCs) are tartrate-resistant acid phosphatase (TRAP)-positive multinuclear cells derived from hematopoietic precursors in the myeloid/monocyte lineage that circulate in the blood after their formation in the bone marrow (17–19). M-CSF and RANKL are the essential factors expressed by osteoblasts, stromal cells and lymphocytes required for OCs formation (20). Due to the strong affinity for bone minerals, BPs can preferentially target OCs in a variety of ways, including effects on OCs differentiation and their recruitment to the bone surface (21), inhibition of OCs adhesion (22), inhibition of OCs activity on the bone surface (23, 24), and shortening of OCs life span by promoting apoptosis (25–30). Denosumab is a monoclonal antibody works by targeting RANKL resulting in RANKL inhibition which further blocks OCs maturation, function and survival ultimately reducing bone resorption (31).

Natural killer (NK) cells are known for their effector functions which include direct natural cytotoxicity, antibody-dependent cellular cytotoxicity (ADCC), as well as secretion of inflammatory cytokines and chemokines that indirectly regulate the functions of other immune cells (32, 33). Immune and bone cells derive from progenitors in the bone marrow, share a common microenvironment and are influenced by similar mediators. RANKL expressed by CD4+ and CD8+ T-cells can induce osteoclastogenesis, providing a link between the immune and skeletal systems. NK cells have also been identified to express RANKL which during their interaction with monocytes can trigger the formation of osteoclasts (34). IFN-γ binds to its receptor on osteoclasts, degrades RANKL signaling and thus inhibits the activation of osteoclasts and protects our bones from being resorbed. IFN-γ is produced predominantly by NK and natural killer T (NKT) cells involved in the innate immune response, and by CD4^+^ Th1 and CD8^+^ cytotoxic T lymphocyte (CTL) effector T cells, once antigen-specific immunity develops (35). Reduced OCs and NK cell function coexist in osteopetrotic mutant rats (36). OCs are capable of secreting a wide range of cytokines and chemokines (37, 38) and activate CD4+ T cells, CD8+ T cells (37) and NK cells (38, 39).

As recommended to develop BRONJ laboratory animal models allowed investigation on the role of inflammatory and immune cells (40), a number of mouse models have been reported (41). While the mouse model provides the essential model to investigate human pathology, it is widely recognized that human and mouse immune systems may differ (42–44). Humanized-BLT (hu-BLT) mice used in this study represents the most advanced and complete humanized mouse model and is the only known humanized mouse model to displays mucosal immunity (45, 46). Generation of hu-BLT mice was performed by surgically implanting pieces of human fetal liver and thymus tissue under the renal capsule of NSG mice, followed by tail vein IV injection of same-donor CD34^+^ hematopoietic cells to support full reconstitution of the human bone marrow (39, 47, 48).

Due to our long-standing studies on ONJ and studying a number of potential mechanisms for the generation and progression of ONJ in different mouse models (38, 49–51), we aimed at identifying factors that may significantly contribute to the induction and progression of ONJ. In a previous study we established epithelial hyperplasia associated with γδ T cells of mouse or human origin in mouse ONJ-like lesions, and based on these studies we concluded that γδ T cells were unlikely cells to mediate the core mechanisms governing the ONJ; however, they may serve as a critical modifier contributing to the different oral mucosal disease variations in ONJ (49). In another study we determined the core function of myeloid cells in the induction of ONJ. In that study CD11b+GR1^hi^ cells in bone marrow and Ly6G+ cells in the oral barrier tissue were depleted, and the development of ONJ-like lesion was significantly attenuated when anti-Ly6G (Gr1) antibody was intraperitoneally injected for 5 days during the second week of tooth extraction, suggesting that local modulation of myeloid cells in the oral barrier tissue may provide the basis for pathogenesis and thus therapeutic as well as preventive strategy for ONJ (50). To determine microenvironmental differences between the bone marrow which contributes to built up of the bone and in oral mucosa where ONJ occurs, we demonstrated that intravenous injection of ZOL in mice induced pro-inflammatory microenvironment in bone marrow and demonstrated significant immune activation and function, however, tooth extraction wound of oral gingival tissues exhibited profound immune suppressive microenvironment associated with dysregulated wound healing due to the effect of ZOL which could potentially be responsible for the pathogenesis of ONJ (38, 51). In examining, the most important potential candidates in ONJ generation we observed significant associations between increased IFN-γ secretion and function within the bone marrow where bone regeneration and increased mineralization occurs, and significant suppression of IFN-γ secretion and gene in oral mucosa where ONJ occurs (38, 51). Therefore, we set the task to investigate a number of parameters including peripheral blood immune cell percentages in human, wild-type mice, and humanized-BLT (hu-BLT) mice, and also investigating the function of immune cells, in particular of NK cell in hu-BLT mice after injections of ZOL and denosumab, which are known to cause ONJ. To our knowledge this is the first report on the role of these two agents on the phenotype and function of NK cells and clear demonstration of suppression of IFN-γ secretion in oral barrier mucosa by these two agents in a relevant mouse model of human disease. We also determined the differential effects of ZOL and denosumab on IFN-γ secretion and NK cell-mediated function in various tissue compartments of hu-BLT mice, demonstrating inverse relationship between bone marrow derived immune cells vs. oral mucosa associated immune cells. These results are extremely crucial in our understanding of mechanisms governing ONJ in oral mucosa and the potential means for novel therapeutic discoveries.

## Materials and methods

### Ethics statement

The UCLA Animal Research Committee reviewed and approved all experimental protocols involving animals (ARC: #1997-136) and procedures were performed in accordance to all federal, state, and local guidelines. Written informed consents approved by UCLA Institutional Review Board (IRB) were obtained from the human blood donors and all the procedures were approved by the UCLA-IRB.

### ZOL or denosumab injection and molar tooth extraction in mice

Combined immunodeficient NOD.CB17-Prkdcscid/J and NOD.Cg-Prkdcscid Il2rgtm1Wjl/SzJ (NSG lacking T, B, and natural killer cells) were purchased from Jackson Laboratory and Humanized-BLT (hu-BLT; human bone marrow/liver/thymus) mice were prepared on NSG background as previously described (47, 52, 53). Seven weeks old female B6 WT and female *Rag2^-/-^* mice (B6(Cg)-*Rag2tm1.1Cgn*/J null mutation in recombination-activating gene-2 resulting in the deficiency of B and T lymphocytes) were purchased from the Jackson Laboratory, Bar Harbor, ME. Mice received a bolus intravenous (IV) injection of 500 µg/Kg ZOL or vehicle 0.9% NaCl solution through retro-orbital venous plexus. One week later, maxillary left first molar was extracted, 5.0 mg/kg carprofen was subcutaneously injected, and mice received 5.0 mg/kg carprofen injection every 24 hours for 48 hours (49). Mice were fed gel food (DietGel Recovery, Clear H2O, Westbrook, ME) for 2 weeks and switched to a conventional mouse pellet food. For denosumab group mice received a bolus intravenous (IV) injection of 120 mg/mice or vehicle 0.9% NaCl solution through retro-orbital venous plexus.

### Human immune cells isolation and generation of human osteoclasts

Human peripheral blood was obtained from healthy donors after the written informed consent approved by UCLA Institutional Review Board (IRB) was obtained. Human NK and monocytes were purified from PBMCs using specific isolation kit (Stem Cell Technologies, Vancouver, Canada). The purity of NK cells and monocytes populations were found to be greater than 90% and 95%, respectively, based on flow cytometric analysis. Monocytes were differentiated to osteoclasts by treating with M-CSF (25 ng/mL) and RANKL (25 ng/mL) for 21 days.

### Mice tissue preparation, and culture of single cell suspension

Hu-BLT, WT and *Rag2^-/-^* mice were euthanized by 100% CO_2_ inhalation on week 2 and week 4 after tooth extraction (n=4 in each group and each time point) followed by a cardiac blood perfusion, and tissues (spleen, bone marrow, pancreas and gingiva from the extraction site) were harvested. The gingiva and pancreas were immediately cut into 1mm^3^ pieces and placed into a digestion buffer containing 1mg/ml collagenase II (gingiva) or collagenase IV (pancreas), 10 U/ml DNAse I, and 1% bovine serum albumin in DMEM and incubated for 20 minutes at 37°C oven with on a 150 rpm shaker. After digestion, the sample was filtered through a 70 µm cell strainer and centrifuged at 1500 rpm for 10 minutes at 4°C. The pellet was re-suspended in DMEM and cells were counted. The spleen was directly mashed and filtered through a 70 µm cell strainer after harvest, and centrifuged for 5 minutes at 1500 rpm at 4°C. The splenic sample was re-suspended in 0.5ml ACK lysis buffer (150mM NH_4_Cl, 1mM KHCO_3_, 0.1mM EDTA) to eliminate red blood cells and incubated for 5 minutes at room temperature followed by centrifugation for 5 minutes at 1500 rpm at 4°C. The pellet was re-suspended in DMEM and cells counted. Bone marrow cells were isolated by flushing femurs with PBS supplemented with 2% heat-inactivated FBS. RPMI 1640 supplemented with 10% Fetal Bovine Serum (FBS), 1% sodium pyruvate, 1% NEAA and 5% antibiotic/antimycotic was used for the tissue cultures. PBMCs were isolated from peripheral blood using Ficoll-Hypaque centrifugation of heparinized blood specimens. The buffy coats containing PBMCs were harvested, washed, and re-suspended in RPMI 1640 medium. Recombinant IL-2 (1000 U/ml) was used to activate the cells for three days (NIH- BRB).

### Surface markers analysis

The dissociated cells were washed and incubated with flow cytometric antibodies (BioLegend, San Diego, CA). IgG2b was used isotype control. After 30 min incubation, the antibody-stained cells were washed and analyzed by flow cytometry (EPICS XL-MCL, Coulter, Miami, FL). The data were evaluated on a computer software package (FlowJo vX, Flowjo, Ashland, OR).

### Enzyme-linked immunosorbent assays and multiplex cytokine assay

Human and mouse IFN-γ ELISA kits were purchased from Biolegend (San Diego, CA) and assay was performed as described by manufacturer. The plates were read in a microplate reader at 450 nm to obtain absorbance value. Multiplex assay was conducted as described in the manufacturer’s protocol for each specified kit. Analysis was performed using a Luminex multiplex instrument (MAGPIX, Millipore, Billerica, MA), and data was analyzed using the proprietary software (xPONENT 4.2, Millipore, Billerica, MA).

### ^51^Cr release cytotoxicity assay

The ^51^Cr release assay was performed as described previously (54). Briefly, different numbers of effector cells were incubated with ^51^Cr–labeled target cells. After a 4-hour incubation period, the supernatants were harvested from each sample and the released radioactivity was counted using the gamma counter. The percentage specific cytotoxicity was calculated as follows:


$$\%\;Cytotoxicity\;=\frac{Experimental\;cpm-spontaneous\;cpm}{Total\;cpm-spontaneous\;cpm}$$

LU 30/10^6^ is calculated by using the inverse of the number of effector cells needed to lyse 30% of target cells _˟_100.

### Antibody-dependent cell-mediated cytotoxicity measurements

Osteoclasts (OCs) were ^51^Cr–labeled and were incubated for an hour, after which unbound ^51^Cr was washed. OCs (1×10^6^ cells/ml) were then left untreated or treated with denosumab (20 μg/ml) or RANKL (25 ng/ml) or a combination of denosumab (20 μg/ml) and RANKL (25 ng/ml) for 30 minutes, and washed with medium to remove excess unbound antibodies. OCs were then cultured with NK cells at various effector to target ratios, and the cytotoxicity against OCs was assessed using the ^51^Cr release cytotoxicity assay as described above.

### Statistical analysis

The data derived from multiple samples per group were presented as the mean ± SEM. All statistical analyses were performed using the GraphPad Prism-9 software. An unpaired or paired, two-tailed student’s t-test was performed for the statistical analysis for experiments with two groups. One-way ANOVA with a Bonferroni post-test was used to compare different groups for experiments with more than two groups. (n) denotes the number of mice for each experimental condition. Duplicate or triplicate samples were used in the *in vitro* studies for assessment. The following symbols represent the levels of statistical significance within each analysis: ****(p value<0.0001), ***(p value 0.0001-0.001), **(p value 0.001-0.01), *(p value 0.01-0.05).

## Results

### Differential proportions of immune cells in peripheral blood of WT mice compared to either hu-BLT mice or humans

**Figure 1:**
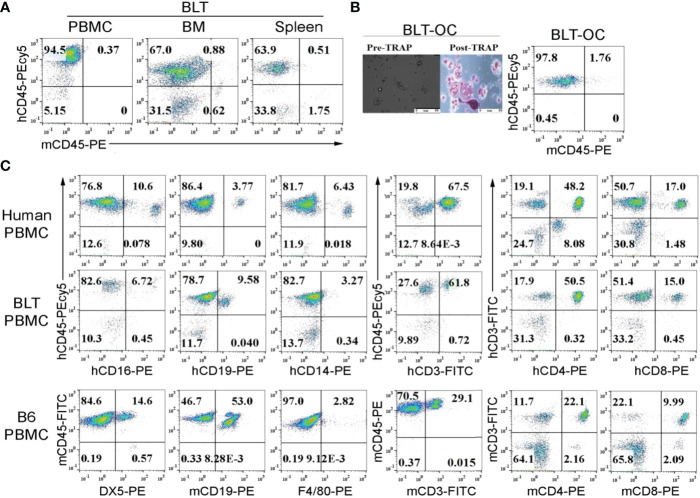
Surface expression of human and mouse CD45 on peripheral blood, bone marrow, splenocytes and bone marrow-derived osteoclasts of hu-BLT mice; comparison of immune cell percentages in human, WT mice and hu-BLT mice PBMCs. Hu-BLT mice acquired humanized hemato-lymphatic system by co-transplantation of human fetal liver and thymus fragments under the renal capsule of NOG/Scid γc-/- immune deficient NSG mice followed by the intravenous injection of autologous CD34+ hematopoietic cells. Eight weeks after tissue implantation, mice were euthanized and tissues were harvested to obtain single cell suspension. PBMCs, bone marrow (BM), and spleen of hu-BLT mice were analyzed for human and mice CD45+ immune cells using flow cytometric analysis **(A)**. Hu-BLT mice BM isolated monocytes were used to generate osteoclasts (OCs) for 21 days as described in Materials and Methods section. On day 21, hu-BLT OCs were washed with 1 X PBS before the pictures were taken by Leica DMI 6000B inverted microscope. Hu-BLT OCs were then treated with Fast Garnet GBC and sodium nitrate (1:1), incubated at 37 degree C for one hour, cells were rinsed and treated with hematoxylin for two mins before pictures were taken by Leica DMI 6000B inverted microscope. Pre- and Post-TRAP-stained pictures are shown in the figure. Hu-BLT OCs were analyzed for human and mice CD45+ immune cells using flow cytometric analysis **(B)**. Immune cell composition was determined in human PBMCs, hu-BLT PBMCs and B6 female WT mice PBMCs using flow cytometric analysis **(C)**.

Hu-BLT mice was found to exhibit 64-95% human-CD45+ (hu-CD45) immune cells in peripheral blood, bone marrow and spleen (**Figure 1A**). Osteoclasts (OCs) generated from the bone marrow (BM) derived monocytes were multinucleated, tartrate-resistant acid phosphate (TRAP) positive, and expressed hu-CD45 surface marker (**Figure 1B**). When compared immune cell composition within the peripheral blood of hu-BLT mice to human and/or WT mouse, we found that the percentages of CD3+, CD3+CD4+ and CD3+CD8+ T cells in hu-BLT PBMCs were very similar to human PBMCs and not WT mouse PBMCs (**Figure 1C**). Lower NK cells and higher B cells percentages were seen in hu-BLT mice PBMCs compared to human PBMCs (**Figure 1C**). Based on previous work from our lab and other labs, it has been established that human PBMCs exhibit higher numbers of T cells and lower numbers of B cells, whereas WT mouse PBMCs have a higher number of B cells and less T cells, as shown in **Figure 1C**. Hu-BLT PBMCs have higher numbers of T cells and less B cells, providing evidence that this mouse model is more like the human system and can be an important tool in studying human diseases.

### ZOL-mediated effect on IFN-γ secretion and NK cell-mediated cytotoxicity in hu-BLT mice

**Figure 2:**
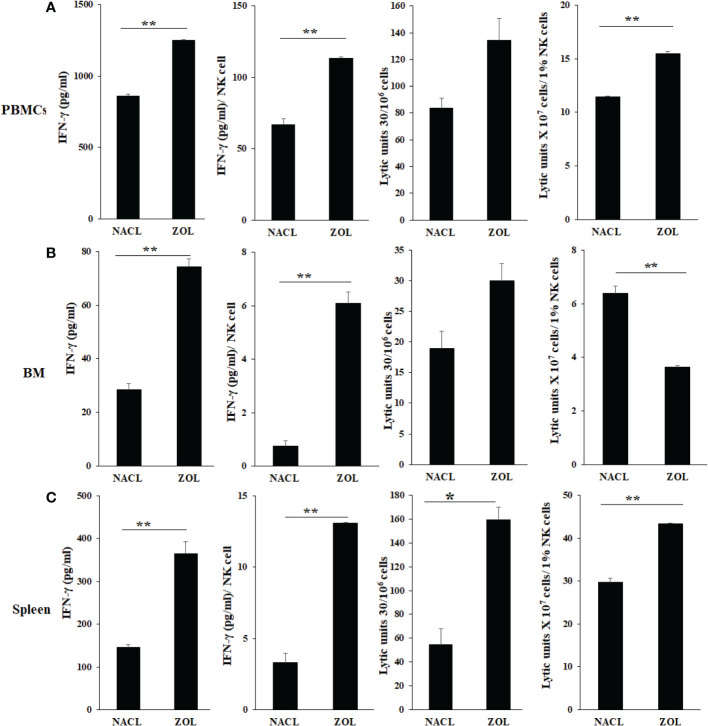
IFN-γ secretion and NK-cell mediated cytotoxicity of PBMCs, BM, and splenocytes of NACL and ZOL-injected hu-BLT mice. Hu-BLT mice were administered with either 0.9% NACL or ZOL (500 µg/kg) *via* IV as described in Materials and Methods section. Four weeks after injections, mice were euthanized and tissues were harvested to obtain single cell suspension. PBMCs **(A)**, BM cells **(B)**, and splenocytes **(C)** of hu-BLT mice were cultured (2 × 10^6^ cells/2ml) with IL-2 (1000 U/ml) for three days, after which the supernatants were harvested and the levels of IFN-γ were determined using specific ELISA. PBMCs **(A)**, BM cells **(B)**, and splenocytes **(C)** were used as effector cells in standard 4-hour ^51^Cr release assay against human OSCSCs tumors. Lytic units (LU) 30/10^6^ cells were determined using inverse number of effector cells required to lyse 30% of OSCSCs × 100. LUs per 1% NK cells were determined using CD16+CD56+ percentages obtained by flow cytometric analysis (n=2) **(A–C)**. **(p value 0.001-0.01), *(p value 0.01-0.05).

To determine the effects of ZOL on the secretion of IFN-γ and NK cell-mediated cytotoxicity, the single cell suspension of peripheral blood, bone marrow, and spleen were cultured in the absence or presence of IL-2 for three days before these functions were determined. ZOL increased IFN-γ secretion and NK cell-mediated cytotoxicity in PBMCs, BM and spleen except NK cell-mediated cytotoxicity per one percent of NK cells was observed lower in ZOL-injected hu-BLT mice BM (**Figure 2**, **Table 1**, and **Figure S1**). We also evaluated the percentages of key immune cell subsets in the peripheral blood, BM and spleen of hu-BLT mice after ZOL injection. ZOL increased CD16+CD56+, and CD3+CD4+ T, and decreased CD3+ T, and CD3+CD8+ T in PBMCs (**Table 2** and **Figure S2A**); increased CD16+CD56+, CD3+ T, and CD3+CD8+ T, and decreased CD3+CD4+ T cells in BM (**Table 2** and **Figure S2B**); increased CD16+CD56+, CD3+ T, and CD3+CD4+ T, and decreased CD3+CD8+ T in spleen (**Table 2** and **Figure S2C**).

Table 1= IFN-γ secreted by splenocytes and PBMCs was compared to IFN-γ secreted by BM of NACL and ZOL-injected hu-BLT mice.

**Table:** 

A: (NACL+ no tooth extraction)			
Bone marrow (n=2)	Spleen (n=2)	Peripheral blood mononuclear cells (n=2)	p values
28.4 (+/- 2.2)	146 (+/- 6)		**
28.4 (+/- 2.2)		858 (+/- 17)	****

**Table:** 

B: (ZOL + no tooth extraction)			
Bone marrow (n=2)	Spleen (n=2)	Peripheral blood mononuclear cells (n=2)	p values
74 (+/-3)	365 (+/- 28)		***
74 (+/-3)		1251 (+/- 5)	****

Hu-BLT mice were administered with either 0.9% NACL (A) or ZOL (500 µg/kg) (B) *via* IV as described in Materials and Methods section. Four weeks after injections, mice were euthanized and tissues were harvested to obtain single cell suspension. BM (n=2), splenocytes (n=2), and PBMCs (n=2) of hu-BLT mice were cultured (2 × 10^6^ cells/2ml) with IL-2 (1000 U/ml) for three days, after which the supernatants were harvested and the levels of IFN-γ were determined using specific ELISA. Statistical analysis was performed to compare the levels of IFN-γ secreted by BM vs. splenocytes or BM vs. PBMCs. ****(p value <0.0001), ***(p value <0.001), **(p value 0.001-0.01).

**Table 2:** Immune cell composition of peripheral blood, bone marrow and spleen of NACL and ZOL-injected hu-BLT mice.

	Peripheral blood mononuclear cells		Bone marrow		Spleen	
Hu-BLT week 4 (n=2)	NACL	ZOL	NACL	ZOL	NACL	ZOL
**% CD16+CD56+**	**7.4**	**9.1**	**2.9**	**8.7**	**1.9**	**3.7**
**% CD3+**	**72**	**58**	**17**	**48**	**28**	**53**
**% CD3+CD4+**	**67**	**88**	**73**	**62**	**77**	**84**
**% CD3+CD8+**	**34**	**9**	**25**	**38**	**24**	**15**

Hu-BLT mice were administered with either 0.9% NACL or ZOL (500 µg/kg) *via* IV as described in Materials and Methods section. Four weeks after injections, mice were euthanized and tissues were harvested to obtain single cell suspension. Immune cell composition was determined in PBMCs (n=2), bone marrow (n=2), and splenocytes (n=2) of hu-BLT mice using flow cytometric analysis.

### Injection of ZOL decreased IFN-γ secretion but increased NK cell-mediated cytotoxicity in tooth extracted oral gingival tissue of hu-BLT mice

**Figure 3:**
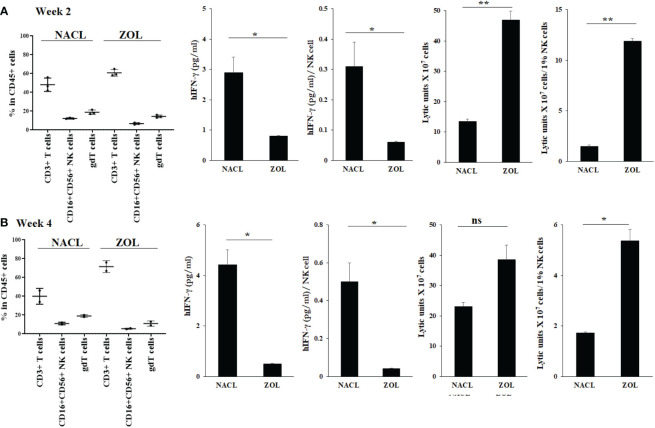
Human CD45+ immune cell percentages, IFN-γ secretion and NK-cell mediated cytotoxicity in oral gingival cells of NACL or ZOL-injected and tooth extracted hu-BLT mice. Hu-BLT mice were administered with either 0.9% NACL or ZOL (500 µg/kg) *via* IV followed by maxillary left first molar extraction as described in Materials and Methods section. Two weeks (n=3) **(A)** and four weeks (n=2) **(B)** after injections, mice were euthanized and oral gingival tissues were harvested to obtain single cell suspension. Surface expression of CD45+CD3+, CD45+CD16+CD56+, and CD45+CD3+gdT+ in oral gingival cells were determined using flow cytometric analysis as described in the Materials and Methods. Oral gingival cells mice were cultured (2 × 10^6^ cells/2ml) with IL-2 (1000 U/ml) for three days, after which the supernatants were harvested and the levels of IFN-γ was determined using specific ELISA. IFN-γ secretion was determined per human CD45+ cells using human CD45+ percentages obtained by flow cytometric analysis. IFN-γ per one NK cell were determined using CD16+CD56+ percentages obtained by flow cytometric analysis. Oral gingival cells were used as effector cells in standard 4-hour ^51^Cr release assay against human OSCSCs tumors. Lytic units (LU) 30/10^6^ cells were determined using inverse number of effector cells required to lyse 30% of OSCSCs × 100. LU per 1% NK cells were determined using CD16+CD56+ percentages obtained by flow cytometric analysis **(A, B)**. **(p value 0.001-0.01), *(p value 0.01-0.05) ns (no significance).

We analyzed the surface receptor expressions for human CD3+ T cells, NK cells (CD16+CD56+) and γδT cells in the gingival tissues recovered from the extraction wound site. Both in week 2 and 4, higher human CD3+ T cells, and less NK and γδT cells were seen in gingival tissue of ZOL-injected as compared to NACL-injected hu-BLT (**Figures 3A, B**, **Figure S3**). Both in week 2 and 4, IFN-γ secretion was found significantly lower in ZOL-injected compared to NACL-injected gingival tissue (**Figures 3A, B**, **S1D**, **Tables 3**, **4** and **Table S3**). NK cell-mediated cytotoxicity against human oral cancer stem cells was significantly higher in ZOL-injected gingival tissue (**Figures 3A, B**).

Table 3IFN-γ secreted per one million hu-CD45+ immune cells or per one NK cell in tissue compartments of NACL or ZOL-injected and tooth-extracted hu-BLT mice.

**Table:** 

A				
IFN-γ/CD45+ cells X 10^6^ Week 2	BLT (NACL+ tooth extraction)	BLT (ZOL + tooth extraction)	Fold change ZOL/NACL	p values row vs. row 3
Bone marrow (n=4)	31 +/- 6	65 +/-9	2.1	**
Spleen (n=4)	131 +/- 9.5	429 +/- 20	3.3	***
Gingiva (n=2)	3 +/- 0.4	0.8 +/- 0.008	0.3	****
Peripheral blood mononuclear cells (n=4)	715 +/- 64	1608 +/- 170	2.25	****
Pancreas (n=3)	7.04 +/- 1	6 +/- 0.8	0.85	ns

**Table:** 

B				
IFN-γ/CD45+ cells X 10^6^ Week 4	BLT (NACL+ tooth extraction)	BLT (ZOL + tooth extraction)	Fold change ZOL/NACL	p values row 2 vs. row 3
Bone marrow (n=4)	45 +/- 7	90 +/- 22	2	**
Spleen (n=4)	180 +/- 12	428 +/- 79	2.4	****
Gingiva (n=2)	4.4 +/- 0.6	0.5 +/- 0.005	0.11	****
Peripheral blood mononuclear cells (n=4)	892 +/- 26	1355 +/- 191	1.5	****
Pancreas (n=3)	8.21 +/- 0.7	8.3 +/- 1.5	1	ns

**Table:** 

C				
IFN-γ/NK Cell Week 2	BLT (NACL+ tooth extraction)	BLT (ZOL + tooth extraction)	Fold change ZOL/NACL	p values row 2 vs. row 3
Bone marrow (n=4)	0.6 +/- 0.15	1.04 +/- 0.23	1.7	*
Spleen (n=4)	5.5 +/- 0.8	13.4 +/- 3	2.4	*
Gingiva (n= 2)	0.31 +/- 0.8	0.06 +/- 0.003	0.2	***
Peripheral blood mononuclear cells (n=4)	89 +/- 11	131 +/- 13	1.5	***
Pancreas (n=3)	0.2 +/- 0.008	0.13 +/- 0.02	0.65	ns

**Table:** 

D				
IFN-γ/NK Cell Week 4	BLT (NACL+ tooth extraction)	BLT (ZOL + tooth extraction)	Fold change ZOL/NACL	p values row 2 vs. row 3
Bone marrow (n=4)	1.1 +/- 0.33	1.9 +/- 1.1	1.7	ns
Spleen (n=4)	7.5 +/- 0.7	11 +/- 3	1.5	*
Gingiva (n=2)	0.5 +/- 0.14	0.04 +/- 0.006	0.08	***
Peripheral blood mononuclear cells(n=4)	113 +/- 6	104 +/- 6	0.9	ns
Pancreas (n=3)	0.2 +/- 0.023	0.4 +/- 0.1	2	*

Hu-BLT mice were administered with either 0.9% NACL or ZOL (500 µg/kg) *via* IV followed by maxillary left first molar extraction as described in Materials and Methods section. Two (A, C) or four (B, D) weeks after tooth extraction, mice were euthanized and tissues were harvested to obtain single cell suspension. BM (n=4), splenocytes (n=4), PBMCs (n=4), oral gingiva (n=2), and pancreas (n=3) of hu-BLT mice were cultured (2 × 10^6^ cells/2ml) with IL-2 (1000 U/ml) for three days, after which the supernatants were harvested and the levels of IFN-γ were determined using specific ELISA. Levels of IFN-γ secreted was determined based on one million human CD45+ immune cells (A, B) or based on one NK cell (C, D). ****(p value <0.0001), ***(p value <0.001), **(p value 0.001-0.01), *(p value 0.01-0.05) ns, no-significance.

Table 4IFN-γ secreted by splenocytes, PBMCs, gingiva, and pancreas was compared to IFN-γ secreted by BM of NACL and ZOL-injected and tooth-extracted hu-BLT mice.

**Table:** 

A: (NACL+ tooth extraction) week 2					
Bone marrow (n=4)	Spleen (n=4)	Peripheral blood mononuclear cells (n=4)	Gingiva (n=2)	Pancreas (n=3)	p values
31 +/-6	131 +/- 9.5				****
31 +/-6		715 +/- 64			****
31 +/-6			3 +/- 0.4		**
31 +/-6				7.04 +/- 1	***

**Table:** 

B: (ZOL + tooth extraction) week 2					
Bone marrow (n=4)	Spleen (n=4)	Peripheral blood mononuclear cells (n=4)	Gingiva (n=2)	Pancreas (n=3)	p values
65 +/- 9	429+/- 20				****
65 +/- 9		1608 +/-170			****
65 +/- 9			0.8 +/-0.008		****
65 +/- 9				6 +/- 0.8	***

**Table:** 

C: (NACL+ tooth extraction) week 4					
Bone marrow (n=4)	Spleen (n=4)	Peripheral blood mononuclear cells (n=4)	Gingiva (n=2)	Pancreas (n=3)	p values
45 +/- 7	180 +/- 12				****
45 +/- 7		892 +/- 26			****
45 +/- 7			4.4 +/- 0.6		*
45 +/- 7				8.21 +/- 0.7	**

**Table:** 

D: (ZOL + tooth extraction) week 4					
BM (n=4)	Spleen (n=4)	Peripheral blood mononuclear cells (n=4)	Gingiva (n=2)	Pancreas (n=3)	p values
90 +/- 22	428 +/- 79				***
90 +/- 22		1355 +/- 191			****
90 +/- 22			0.5 +/- 0.005		**
90 +/- 22				8.3 +/- 1.5	**

Hu-BLT mice were administered with either 0.9% NACL (A, C) or ZOL (500 µg/kg) (B, D) *via* IV followed by maxillary left first molar extraction as described in Materials and Methods section. Two (A, B) or four (C, D) weeks after tooth extraction, mice were euthanized and tissues were harvested to obtain single cell suspension. BM (n=4), splenocytes (n=4), PBMCs (n=4), oral gingiva (n=2), and pancreas (n=3) of hu-BLT mice were cultured (2 × 10^6^ cells/2ml) with IL-2 (1000 U/ml) for three days, after which the supernatants were harvested and the levels of IFN-γ were determined using specific ELISA. Levels of IFN-γ secreted was determined based on human CD45+ immune cells, and statistical analysis was performed to compare the levels of IFN-γ secreted by BM vs. splenocytes or BM vs. PBMCs. ****(p value <0.0001), ***(p value <0.001), **(p value 0.001-0.01), *(p value 0.01-0.05).

### Effect of ZOL injection on IFN-γ secretion and key immune cells in PBMCs, BM, spleen and pancreas of tooth-extracted hu-BLT mice

**Figure 4:**
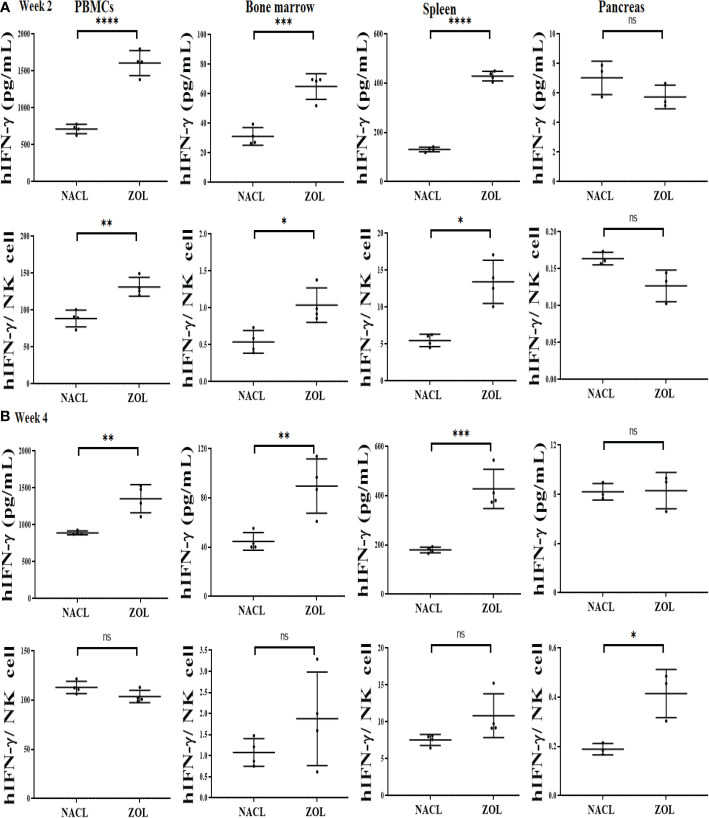
IFN-γ secretion in PBMCs, BM, spleen and pancreas of NACL or ZOL-injected and tooth-extracted hu-BLT mice. Hu-BLT mice were administered with either 0.9% NACL or ZOL (500 µg/kg) *via* IV followed by maxillary left first molar extraction as described in Materials and Methods section. Two **(A)** or four weeks **(B)** after tooth extraction, mice were euthanized and tissues were harvested to obtain single cell suspension. PBMCs (n=4), BM (n=4), splenocytes (n=4), and pancreas (n=3) of hu-BLT mice were cultured (2 × 10^6^ cells/2ml) with IL-2 (1000 U/ml) for three days, after which the supernatants were harvested and the levels of IFN-γ were determined using specific ELISA. IFN-γ secretion per human CD45+ cell was determined using CD45 percentages obtained by flow cytometric analysis. IFN-γ per one NK cell was determined using CD16+CD56+ percentages obtained by flow cytometric analysis **(A, B)**. ****(p value<0.0001), ***(p value<0.001), **(p value 0.001-0.01), *(p value 0.01-0.05), ns (no significance).

We determined the effect of ZOL in combination with tooth extraction on IFN-γ secretion, and found higher IFN-γ secretion in PBMCs, bone marrow and spleen of the ZOL-injected hu-BLT mice in week 2 and 4 after tooth extraction (**Figures 4A, B**, **Tables 3**, **4**, and **Figures S4, S5**). In pancreas, IFN-γ secretion was found higher both of week 2 and 4 (**Figure S4**), IFN-γ secretion per hu-CD45+ immune cells and per one NK cell were found lower on week 2 (**Figure 4A** and **Table 3**), but on week 4 similar levels were seen for IFN-γ secretion per hu-CD45+ immune cells and higher per one NK cell in ZOL-injected hu-BLT mice (**Figure 4B** and **Table 3**). In both week 2 and 4, NACL and ZOL injected mice, IFN-γ secretion level order was PBMCs > spleen > BM > pancreas > gingiva (**Table 4**). The secretion levels of TNF-α and IL-6 were found to be higher in PBMCs, bone marrow and spleen of the ZOL-injected hu-BLT mice in week 2 and 4 (**Figure S5**). Next, we evaluated the percentages of key immune cell subsets in the peripheral blood, BM, spleen, and pancreas of ZOL and NACL injected tooth-extracted hu-BLT mice. On week 2, ZOL-injected tooth-extracted hu-BLT PBMCs and spleen showed increased CD16+CD56+, and CD3+ T; BM showed increased CD16+CD56+, and decreased CD3+ T cells; pancreas showed similar CD16+CD56+, and increased CD3+ T (**Table S4**, and **Figure S6**). On week 4, ZOL-injected tooth-extracted hu-BLT PBMCs showed increased CD16+CD56+, CD3+CD4+ T, and decreased CD3+ T, and CD3+CD8+ T cells; BM showed increased CD16+CD56+, CD3+ T, and CD3+CD8+ T, and decreased CD3+CD4+ T; spleen increased CD16+CD56+, CD3+ T, and CD3+CD4+ T, and decreased CD3+CD8+ T cells; pancreas increased CD16+CD56+, CD3+ T, CD3+CD4+ T, and decreased CD3+CD8+ T cells (**Table 5**, and **Figure S6**).

**Table 5:** Percentages of immune cells in PBMCs, BM, splenocytes and pancreas of NACL or ZOL-injected and tooth-extracted hu-BLT mice.

Hu-BLT week 4 (n=4)	Peripheral blood mononuclear cells		Bone marrow		Spleen		Pancreas	
	NACL	ZOL	NACL	ZOL	NACL	ZOL	NACL	ZOL
**% CD16+CD56+**	**6**	**8.3**	**2.7**	**5.1**	**2.3**	**4.04**	**2.3**	**5.2**
**% CD3+**	**68**	**55.3**	**16.5**	**30.5**	**28**	**51.3**	**40.5**	**60.3**
**% CD3+CD4+**	**63**	**78.5**	**66**	**54**	**74**	**85**	**20**	**25**
**% CD3+CD8+**	**37**	**21.5**	**35**	**46**	**26.3**	**15**	**80**	**75**

Hu-BLT mice were administered with either 0.9% NACL or ZOL (500 µg/kg) *via* IV followed by maxillary left first molar extraction as described in Materials and Methods section. Four weeks after tooth extraction, mice were euthanized and tissues were harvested to obtain single cell suspension. Immune cell composition was determined in PBMCs (n=4), BM (n=4), splenocytes (n=4), and pancreas (n=3) of hu-BLT mice using flow cytometric analysis.

### Effect of ZOL injection on NK cell-mediated cytotoxicity in PBMCs, BM, spleen and pancreas of tooth-extracted hu-BLT mice

**Figure 5:**
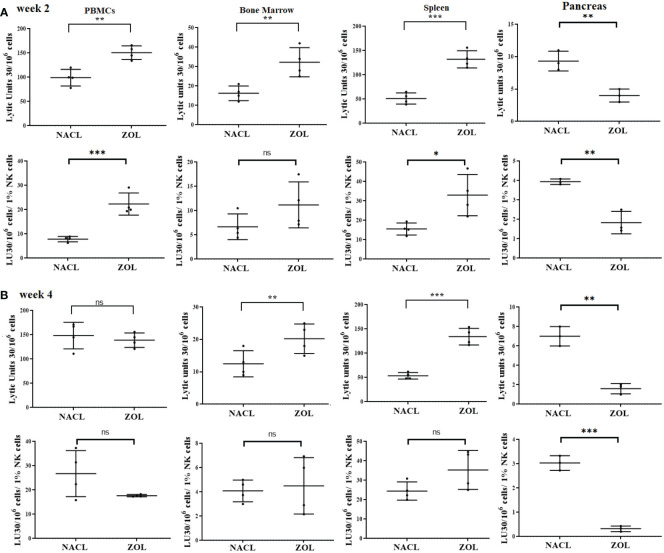
NK cell-mediated cytotoxicity in PBMCs, BM, spleen and pancreas of NACL or ZOL-injected and tooth-extracted hu-BLT mice. Hu-BLT mice were administered with either 0.9% NACL or ZOL (500 µg/kg) *via* IV followed by maxillary left first molar extraction as described in Materials and Methods section. Two **(A)** or four weeks **(B)** after tooth extraction, mice were euthanized and tissues were harvested to obtain single cell suspension. PBMCs (n=4), BM (n=4), splenocytes (n=4), and pancreas (n=3) of hu-BLT mice were cultured (2 × 10^6^ cells/2ml) with IL-2 (1000 U/ml) for three days, after which the cells were used as effector cells in standard 4-hour ^51^Cr release assay against human OSCSCs tumors. Lytic units (LU) 30/10^6^ cells were determined using inverse number of effector cells required to lyse 30% of OSCSCs × 100. LUs per 1% NK cell were determined using CD16+CD56+ percentages obtained by flow cytometric analysis **(A, B)**. ***(p value<0.001), **(p value 0.001-0.01), *(p value 0.01-0.05), ns (no significance).

On week 2, NK cell-mediated cytotoxicity against human oral cancer stem cells was higher in PBMCs, bone marrow and spleen, and lower in pancreas of ZOL-injected hu-BLT mice (**Figure 5A**). On week 4, NK cell-mediated cytotoxicity against human oral cancer stem cells was slightly lower in PBMCs, but continued to be higher in bone marrow and spleen, and significantly lower in pancreas in ZOL-injected hu-BLT (**Figure 5B**).

### Denosumab-mediated effect in IFN-γ secretion and NK cell-mediated cytotoxicity in hu-BLT mice

**Figure 6:**
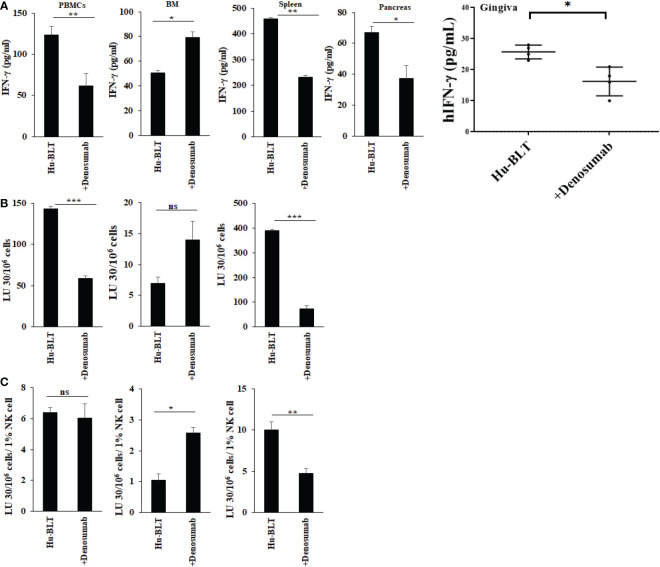
IFN-γ secretion and NK-cell mediated cytotoxicity in NACL and denosumab-injected hu-BLT mice. Hu-BLT mice were administered with either 0.9% NACL or denosumab (120 mg/mice) *via* IV as described in Materials and Methods section. Four weeks after injections, mice were euthanized and tissues were harvested to obtain single cell suspension. PBMCs (n=2), BM cells (n=2), splenocytes (n=2), pancreas (n=2), and gingiva (n=3) of hu-BLT mice were cultured (2 × 10^6^ cells/2ml) with IL-2 (1000 U/ml) for three days, after which the supernatants were harvested and the levels of IFN-γ were determined using specific ELISA **(A)**. PBMCs (n=2), BM cells (n=2), and splenocytes (n=2) were used as effector cells in standard 4-hour ^51^Cr release assay against human OSCSCs tumors. Lytic units (LU) 30/10^6^ cells were determined using inverse number of effector cells required to lyse 30% of OSCSCs × 100 **(B)**. LUs per 1% NK cells were determined using CD16+CD56+ percentages obtained by flow cytometric analysis (n=2) **(C)**. ***(p value 0.0001-0.001), **(p value 0.001-0.01), *(p value 0.01-0.05), ns (no significance).

To determine the effects of denosumab on the secretion of IFN-γ and NK cell-mediated cytotoxicity, the single cell suspension of peripheral blood, bone marrow, spleen, pancreas and gingiva were treated with IL-2 for three days before these functions were determined. Higher levels of IFN-γ secretion were observed in BM, but decreased in PBMCs, spleen, pancreas, and gingiva of denosumab-injected hu-BLT mice (**Figure 6A**). NK cell-mediated cytotoxicity was also increased in BM but decreased in PBMCs and spleen of denosumab-injected hu-BLT mice (**Figures 6B, C**). Denosumab increased CD3+CD8+, and decreased CD3+, and CD3+CD4+ T in PBMCs (**Figure S7A**); increased CD16+CD56+, CD3+ CD8+ T and decreased CD3+CD4+ T in BM (**Figure S7B**); increased CD3+CD8+ T, and CD16+CD56+, CD3+, and CD3+CD4+ T cells in spleen (**Figure S7C**).

### Higher levels of NK cell-mediated ADCC were seen against OCs treated with a combination of denosumab and RANKL

**Figure 7:**
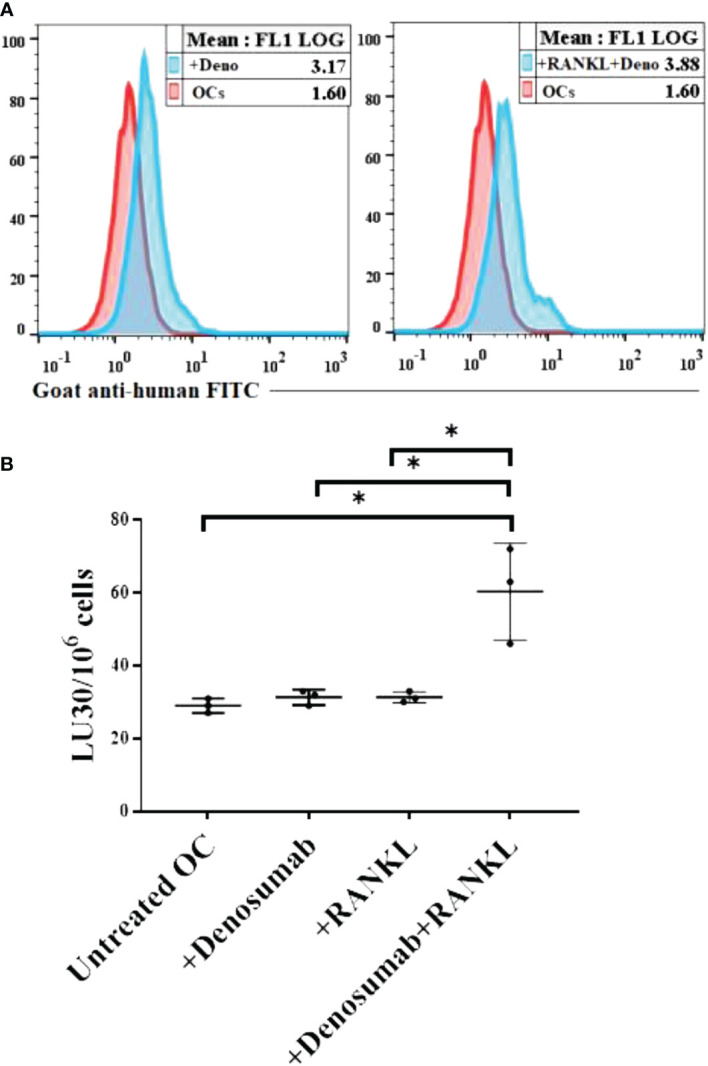
Higher levels of NK cell-mediated ADCC was seen against OCs treated with a combination of denosumab and RANKL. Human OCs were generated as described in the Materials and Methods. OCs were left untreated or treated with denosumab (20 μg/ml) or RANKL (25 ng/ml) or a combination of denosumab (20 μg/ml) and RANKL (25 ng/ml) for 30 minutes. The unbounded antibodies were washed away, and the surface expression levels were analyzed after cells were stained with goat anti-human FITC using flow cytometry. IgG2 isotype antibodies were used as controls. One of three representative experiments is shown in the figure **(A)**. OCs were generated as described in the Materials and Methods. Purified NK cells (1×10^6^ cells/ml) from healthy individuals were treated with IL-2 (1000 U/ml) for 18 hours and were as effectors in chromium release assay. OCs were labeled with ^51^Cr for an hour after which cells were washed to remove unbound ^51^Cr. ^51^Cr-labeled OCs were then left untreated or treated with denosumab (20 μg/ml) or RANKL (25 ng/ml) or a combination of denosumab (20 μg/ml) and RANKL (25 ng/ml) for 30 minutes. The unbounded antibodies were washed away, and the cytotoxicity against the OCs was determined using a standard 4-6 hour ^51^Cr release assay. The lytic units (LU) 30/10^6^ cells were determined using the inverse number of NK cells required to lyse 30% of OCs × 100 (n=3) **(B)**. *(p value 0.01-0.05).

We treated human OCs with denosumab alone or with a combination of denosumab and RANKL followed by surface analysis using flow cytometer (**Figure 7A**). Later, we determined the antibody dependent cell-mediated cytotoxicity (ADCC) using untreated or denosumab or RANKL or a combination of denosumab and RANKL treated human OCs as targets and IL-2 treated human NK cells as effectors. Although no ADCC was seen when OCs were with denosumab or RANKL alone, but significant levels of ADCC was seen when OCs were treated with a combination of denosumab and RANKL (**Figure 7B**).

## Discussion

Many attempts were made to delineate the underlying mechanisms of osteonecrosis of the jaw (ONJ) in patients receiving either bisphosphonates or denosumab previously. Our findings indicate that gingiva unlike peripheral blood, bone marrow, or spleen have different dynamics of immune function and regulation. Although we have observed increased immune function by bisphosphonate ZOL and denosumab in BM, both in terms of NK cell-mediated cytotoxicity and secretion of IFN-γ, other tissue compartments such as PBMCs, spleen and gingiva have distinct profiles. Both PBMCs and spleen were able to exhibit higher NK cell-mediated cytotoxicity and secretion of IFN-γ when activated by ZOL in comparison to NACL, but the injection of denosumab exhibited lower NK cell-mediated cytotoxicity and secretion of IFN-γ in PBMCs and spleen when compared to NACL in hu-BLT mice, suggesting different mechanisms of action in the peripheral tissues between ZOL and denosumab, ultimately leading to the induction of ONJ. However, in both cases the gingival immune cells had much lower ability to increase IFN-γ secretion. Indeed, ZOL injection delayed wound healing after tooth extraction in tooth socket of hu-BLT mice after 4 weeks of treatment suggestive of ONJ (manuscript in prep), whereas NACL injection did not exhibit such aberrations in the tooth socket after tooth extraction. Upon histological examination, on week 4 after tooth extraction and injection of ZOL many inflammatory cells were remaining in the connective tissue immediately adjacent to the bone with larger and prominent areas of necrotic bone, whereas in NACL injected mice the wound had already resolved, with very few inflammatory cells remaining in the connective tissue and a lack of necrotic bone lesions (manuscript in prep). Such abnormal manifestations by ZOL within the tooth socket could be due to the abnormal induction of immune activation, in particular suppression of IFN-γ secretion and elevated NK cell-mediated cytotoxicity adjacent to bone as detailed in our studies.

There are a number of differences between the WT mice and hu-BLT mice in terms of their response to ZOL activation before and after tooth extraction. WT mice has in general higher percentages of B cells and lower percentages of T cells whereas the proportions of immune cell subsets are similar between the human PBMCs and reconstituted hu-BLT PBMCs with some slight differences in the proportions of NK cells in hu-BLT mice in comparison to humans (55) (**Figure 1C**). In three different mice models of WT, *Rag2^-/-^* and hu-BLT, four weeks after tooth extraction, the fold increase of IFN-γ secretion in BM cells by ZOL vs. NACL treatment was 4.85 for WT, 1.4 for *Rag2^-/-^* and 2.7 for hu-BLT mice. Therefore, BM cells of WT mice had higher ability to upregulate IFN-γ secretion with ZOL whereas *Rag2^-/-^* mice had the lowest ability and hu-BLT mice was intermediate. When examining the ratio of spleen to BM, WT mice without any injection or tooth extraction had 1.83 fold increase whereas with tooth extraction and NACL injection it was 1.7 fold and with ZOL injection it was 0.92 (**Table S1**). In *Rag2^-/-^* mice the fold increase of spleen to BM was 1.19, 1, and 0.375 for no tooth extraction in the absence of injection, and tooth extraction with NACL injection, and tooth extraction with ZOL injection, respectively (**Table S2**). These results indicated that the higher fold of activation and release of IFN-γ in BM cells resulted in lower upregulation of splenocytes in terms of IFN-γ secretion. In contrast the differences between spleen and BM for hu-BLT mice was 8.06 for no tooth extraction and no injection, and the levels decreased to 4.98 and 5.45 fold in week 2 of tooth extraction with NACL and ZOL injection, respectively. The fold differences of IFN-γ between spleen and BM for hu-BLT mice four weeks after tooth extraction was 4.97 with NACL and 3.98 with ZOL (**Table S3**). These results suggested that if BM cells receive no or lower signals to increase IFN-γ, the periphery will have capability to increase IFN-γ at an increased level. Indeed, PBMCs from the hu-BLT without tooth extraction or injection has ability to upregulate 53 fold when compared to BM cells and that fold decreased to 15.9 and 11.82 for week 2 extractions with NACL and ZOL injections, respectively, and 13.2 and 9.33 for week 4 tooth extractions with NACL and ZOL, respectively, again emphasizing the observation that if BM immune cells receive no or lower signals to increase IFN-γ, immune cells within the peripheral blood and tissues will be able to increase IFN-γ at a much higher levels. Therefore, the higher the increase in BM cells, the lower will be the activation in the periphery. These observations may go beyond the effect of ZOL and denosumab, and implicate other activating agents/signals in which they are involved in the stimulation or priming of the BM, thus affecting the magnitude of increase in immune function in the periphery, or even play a major role in the ultimate effectiveness of peripheral immune cells in elimination or control of infections and malignancies. Therefore, signaling and activation of immune effectors in BM is likely to affect the levels and activation of the immune function in the peripheral blood and tissues.

We have also observed higher percentages of NK cells almost in all tissues of hu-BLT mice after ZOL injections regardless of either it was with tooth extraction or no tooth extraction. NK cell-mediated cytotoxicity and secretion of IFN-γ was higher for BM, spleen and PBMCs after ZOL injection when compared to NACL, whereas pancreas showed decreased NK cell-mediated cytotoxicity and similar or increased secretion of IFN-γ with ZOL. In gingiva, we observed significantly less IFN-γ after ZOL injection but higher NK cell-mediated cytotoxicity. In our previous paper, we have shown that ZOL injection in WT and *Rag2^-/-^* mice resulted in a severe decrease in IFN-γ secretion in the gingiva and the higher increase in BM resulting in higher suppression in the gingiva (51). Therefore, although we see variable results in secretion of IFN-γ in BM, spleen and PBMCs, we always see suppression in the gingiva in WT, *Rag2^-/-^*, or hu-BLT mice. Similar results were also observed when denosumab was injected in hu-BLT mice. In no tooth extraction model injection of denosumab increased the levels of IFN-γ secretion in BM but decreased in other tissues we tested, with gingiva having the most severe decreases. The fold increase of IFN-γ secretion when spleen was compared with BM in the absence of denosumab injection was 8.5 fold, but in the presence of denosumab injection it was 3.12. Based on these results, it appears that denosumab is more suppressive for the peripheral tissues when compared to ZOL. Indeed, in all the peripheral tissues, including PBMCs, splenocytes, pancreas and gingiva the fold increase to BM were all lower. Thus, the dynamics of NK cells’ activation by ZOL and denosumab in BM and peripheral tissues are distinct in hu-BLT mice, although both lead to suppression of IFN-γ secretion in gingiva. It is likely that different mechanisms are used for NK cells suppression by these two drugs. In both cases if stromal cells are unable to undergo differentiation and retain their stem like phenotype, it is likely that they will be targeted by the NK cells leading to ONJ, since stem cells are major targets of NK cells (56).

Increased NK cell-mediated cytotoxicity in the presence of decreased IFN-γ secretion in gingiva after ZOL and denosumab injection may suggest that NK cells may be cytotoxic to stromal cells which have not received signals to undergo differentiation due to the lack of IFN-γ secretion from the NK cells since IFN-γ is an important cytokine to drive differentiation of the cells (57, 58). At present it is not clear why the gingival NK cells show decreased IFN-γ secretion, however, as indicated above, if BM-derived NK cells receive higher signals and are primed and activated within the BM, which we have shown in three different mouse models, depending on the quality and quantity of signals that they receive in the periphery, this may determine their ultimate fate in the gingiva. However, it is not clear why gingival NK cells retain their cytotoxic function but lose ability to secrete IFN-γ. This is the opposite of split anergy in NK cells, which we have coined previously to indicate the function of NK cells which have lost ability to kill stem cells but retained the ability to secrete cytokines (59). It is conceivable to think that NK cells may contain preformed granules that upon contact can deliver to the targets even in situations where they are unable to proliferate or secrete cytokines. These scenarios should await future investigations.

Although the percentages of the NK cells are lower in the gingiva after ZOL treatment (on average 12.2% for NACL injected mice and 6.8% for ZOL injected mice for week 2 after extraction, and 11% and 5.5% for NACL and ZOL injected mice respectively on Week 4 after tooth extraction), they still exhibited increased cytotoxicity but lower secretion of IFN-γ, suggesting that these cells are locally primed and activated, however, their activation may be limited since there is a suppression of IFN-γ secretion. Since T cell activation can also contribute to IFN-γ secretion, the sum of suppression in T and NK cells and potentially other immune subtypes may contribute to the overall suppression of IFN-γ in the gingival tissues. Suppression of IFN-γ secretion in gingival cells could be due to activation induced cell death leading to immunosuppression, since the local microenvironment in the oral cavity is laden with bacteria, food particles etc. that may over activate, already highly primed immune cells by the ZOL or denosumab. Therefore, over activation of gingival immune cells with the sum of all activating signals may be the cause of exhaustion, senescence and activation induced cell death resulting in immune suppression. Indeed, in WT, *Rag2^-/-^* and hu-BLT mice gingiva exhibited significant suppression of IFN-γ secretion, whereas it was variable in PBMCs, spleen and pancreas depending on the mouse model. In addition, all the three mouse models tested in this study also have in common activation of bone marrow cells with ZOL and denosumab. Therefore, studies from WT mice should be interpreted cautiously, when extrapolating to human disease since the dynamics of immune activation in the peripheral tissues are very different between the WT and hu-BLT mice. *Rag2^-/-^* mice has the most severe suppression of the IFN-γ in the gingival tissues, and the highest activation of cells within the BM and other peripheral tissues (51).

To understand the mechanisms by which denosumab can increase bone formation resulting in the reversal or decrease in progression of osteoporosis, we undertook studies to determine the potential mechanisms by which this antibody may act on osteoclasts. Differentiated OCs present receptors for RANK on the surface and are susceptible to antibody dependent cellular cytotoxicity when denosumab is added in the presence of RANKL. It appears that in the absence of RANKL, denosumab does not increase ADCC substantially, indicating that RANKL should be provided by other immune effectors in the microenvironment and that OCs themselves may not produce high levels of RANKL. RANKL is known to be provided by a number of lymphocytes such as T and NK cells (60, 61). Therefore, denosumab mediated effect could be due to the decrease in the bone resorptive function of osteoclasts due to the induction ADCC and killing of OCs, whereas ZOL mediated effects on OCs cell death could be due to a completely different mechanism.

IFN-γ can function both as a pro- or anti-resorptive cytokine, but the reasons for why IFN-γ has variable effects in bone is unknown. IFN-γ was shown to have both direct anti-osteoclastogenic and indirect pro-osteoclastogenic properties *in vivo* (62). It has been shown that IFN-γ blunts OCs formation through direct targeting of OCs precursors but indirectly stimulates OCs formation and promotes bone resorption by stimulating antigen-dependent T cell activation and T cell secretion of the osteoclastogenic factors RANKL and TNF-α. Thus, the effect of IFN-γ on OCs are variable and may dependent on their maturational stages. However, IFN-γ has many important functions on other cells including osteoblasts and immune cells and, proper induction of this cytokine is imperative for the functioning of the immune cells and osteoblasts in addition to OCs. Suppression of IFN-γ in gingiva may be responsible for the inhibition of immune cells function, subsequently leading to dysregulated osteoblastic and osteoclastic activities. Restoration of IFN-γ in the local microenvironment may result in establishment of homeostatic balance in the gingiva and prevention of ONJ.

OCs are important regulators of osteoblasts and balanced functioning osteoclast/osteoblast is imperative for bone health. We have previously shown that OCs are important and are key activators of the NK cells. Therefore, OCs have significant capacity to modulate the function of the immune cells, changing the fate of osteoblast differentiation and function. The interplay between the CD4+ or CD8+ T cells and γδ T cells and NK cells and OCs within the bone microenvironment is currently being unraveled and future investigations should focus on the interaction of these cells within the bone microenvironment.

Similar to our studies, previous studies in patients with multiple myeloma indicated decrease in the levels of TGF-β, VEGF and a number of other cytokines in BRONJ patients (63). One of the limitations of our studies is the lack of comparative studies between patients who suffer from ONJ and our results from humanized mice. Although our model of study ONJ in hu-BLT does not contain cancer, there are some parallels with human cancer patients in which there are lower percentages of NK cells in hu-BLT mice (**Figure 1C**) similar to patients with cancer (55, 64–67). Such similarities may provide the rationale for the extension of studies from hu-BLT mice to humans with ONJ. However, for the sake of direct comparisons we intend to implant tumors in hu-BLT mice and study the effect of ZOL and Denosumab in the setting of cancer in our future experiments.

## Data availability statement

The original contributions presented in the study are included in the article/**Supplementary Material**. Further inquiries can be directed to the corresponding authors.

## Ethics statement

The studies involving human participants were reviewed and approved by UCLA Institutional Review Board (IRB). The patients/participants provided their written informed consent to participate in this study.

## Author contributions

KaK performed all the experiments, analyzed the results, and wrote the manuscript. YS, KeK, KM, and AH assisted KaK in *in-vivo* work. IN was the co-principal investigator, obtained the funding and designed the study. AJ was the principal investigator, obtained the funding, designed the study, and wrote the manuscript along with first author. All authors contributed to the article and approved the submitted version.
