# Supplementary material for: Augmentation of IFN-γ by bone marrow derived immune cells in the presence of severe suppression of IFN-γ in gingivae induced by zoledronic acid and denosumab in Hu-BLT mice model of ONJ
Source: Front Endocrinol (Lausanne). 2023 Jan 20;14:1111627. doi: 10.3389/fendo.2023.1111627 (PMC9895394; doi:10.3389/fendo.2023.1111627)
Supplement: Supplementary file 1 [file DataSheet_1.docx]

**Supplementary Data**

**Severe *in vivo* suppression of IFN-γ in gingivae induced by Zoledronic acid and Denosumab; a potential mechanism for the osteonecrosis of the jaw (ONJ)**

Kawaljit Kaur ^1,2^, Yujie Sun ^1,3^, Keiichi Kanayama ^1,3,4^, Kenzo Morinaga ^1,3,5^, Akishige Hokugo ^1,6^, Ichiro Nishimura ^1,2,3*^ and Anahid Jewett ^1,2*^

^1^ Weintraub Center for Reconstructive Biotechnology, ^2^ Division of Oral Biology & Medicine, ^3^ Division of Advanced Prosthodontics UCLA School of Dentistry, Los Angeles, CA 90095 USA

^4^ Department of Periodontology, Asahi University School of Dentistry, Gifu, 501-0296, Japan

^5^ Department of Oral Rehabilitation, Fukuoka Dental College, Fukuoka, 814-0175, Japan

^6^ Division of Plastic Surgery, David Geffen School of Medicine at UCLA, Los Angeles, CA 90095, USA

^*^**Corresponding authors:**

Anahid Jewett, UCLA School of Dentistry, 10833 Le Conte Ave., Los Angeles, CA 90095, USA; email: [ajewett@ucla.edu](mailto:ajewett@ucla.edu); Ph: 1-310-794-7612; Fax: 1-310-825-3210

Ichiro Nishimura; Weintraub Center for Reconstructive Biotechnology, UCLA School of Dentistry, Box 951668, CHS B3-087, Los Angeles, CA 90095, USA; email: [inishimura@dentistry.ucla.edu](mailto:inishimura@dentistry.ucla.edu), **Tel:** 1-310-206-3970, **Fax:** 1-310-794-7109

**Table legends**

**Table S1: IFN-γ secreted by tissues was compared to IFN-γ secreted by BM in NACL and ZOL-injected WT b6 female mice with/without tooth extraction**

WT mice were administered with either 0.9% NACL or ZOL (500 µg/kg) via tail vein followed by no tooth extraction or maxillary left first molar extraction as described in Materials and Methods section. Four weeks after injections, mice were euthanized and tissues were harvested to obtain single cell suspension for analysis. Cells were cultured (2 × 10^6^ cells/2ml) with IL-2 (10000 U/ml) for five days, after which the supernatants were harvested and the levels of IFN-γ were determined using specific ELISA. Levels of IFN-γ secreted by BM were compared with other tissues as shown in tables.

**Table S2: IFN-γ secreted by tissues was compared to IFN-γ secreted by BM in NACL and ZOL-injected *Rag2^-/-^*** **mice with/without tooth extraction**

*Rag2^-/-^* mice were administered with either 0.9% NACL or ZOL (500 µg/kg) via tail vein followed by no tooth extraction or maxillary left first molar extraction as described in Materials and Methods section. Four weeks after injections, mice were euthanized and tissues were harvested to obtain single cell suspension for analysis. Cells were cultured (2 × 10^6^ cells/2ml) with IL-2 (10000 U/ml) for five days, after which the supernatants were harvested and the levels of IFN-γ were determined using specific ELISA. Levels of IFN-γ secreted by BM were compared with other tissues as shown in tables.

**Table S3: IFN-γ secreted by tissues was compared to IFN-γ secreted by BM in NACL and ZOL-injected hu-BLT** **mice with/without tooth extraction.**

Hu-BLT mice were administered with either 0.9% NACL or ZOL (500 µg/kg) via tail vein followed by no tooth extraction or maxillary left first molar extraction as described in Materials and Methods section. Four weeks after injections, mice were euthanized and tissues were harvested to obtain single cell suspension for analysis. Cells were cultured (2 × 10^6^ cells/2ml) with IL-2 (10000 U/ml) for five days, after which the supernatants were harvested and the levels of IFN-γ were determined using specific ELISA. Levels of IFN-γ secreted by BM were compared with other tissues as shown in tables.

**Table S4: Percentages of immune cells in PBMCs, BM, splenocytes and pancreas of NACL or ZOL-injected and tooth-extracted hu-BLT mice**

Hu-BLT mice were administered with either 0.9% NACL or ZOL (500 µg/kg) via IV followed by maxillary left first molar extraction as described in Materials and Methods section. Two weeks after tooth extraction, mice were euthanized and tissues were harvested to obtain single cell suspension. Immune cell composition was determined using flow cytometric analysis.

**Table S1**

| **WT (No tooth extraction)** | **BM**  (n=9) | **Spleen**  (n=9) | **Gingiva**  (n=5) | **Pancreas**  (n=5) | **p-values** |
| --- | --- | --- | --- | --- | --- |
|  | 154 (+/-35) | 282 (+/-168) |  |  | ** |
|  | 154 (+/-35) |  | 121 (+/-45) |  | ns |
|  | 154 (+/-35) |  |  | 98 (+/-29) | *** |

| **WT (NACL+ tooth extraction)** | **BM**  (n=5) | **Spleen**  (n=5) | **Gingiva**  (n=4) | **p-values** |
| --- | --- | --- | --- | --- |
|  | 182 (+/-63) | 310 (+/-121) |  | *** |
|  | 182 (+/-63) |  | 119 (+/-28) | *** |

| **WT (ZOL+ tooth extraction)** | **BM**  (n=5) | **Spleen**  (n=5) | **Gingiva**  (n=4) | **p-values** |
| --- | --- | --- | --- | --- |
|  | 884 (+/-437) | 810 (+/-224) |  | ns |
|  |  |  | 45 (+/-2) | **** |

**Table S2**

| ***Rag2^-/-^* (No tooth extraction)** | **BM**  (n=9) | **Spleen** (n=9) | **Gingiva**  (n=5) | **Pancreas**  (n=5) | **Adipose**  (n=5) | **p-values** |
| --- | --- | --- | --- | --- | --- | --- |
|  | 679 (+/-164) | 812 (+/-320) |  |  |  | * |
|  | 679 (+/-164) |  | 129 (+/-39) |  |  | **** |
|  | 679 (+/-164) |  |  | 379 (+/-105) |  | *** |
|  | 679 (+/-164) |  |  |  | 556 (+/-125) | * |

| ***Rag2^-/-^* (NACL+ tooth extraction)** | **BM**  (n=9) | **Spleen**  (n=8) | **Gingiva**  (n=8) | **p-values** |
| --- | --- | --- | --- | --- |
|  | 457 (+/-52) | 457 (+/-138) |  | ns |
|  |  |  | 23 (+/-17) | **** |

| ***Rag2^-/-^* (ZOL+ tooth extraction)** | **BM**  (n=9) | **Spleen**  (n=9) | **Gingiva**  (n=8) | **p-values** |
| --- | --- | --- | --- | --- |
|  | 624 (+/-139) | 234 (+/-84) |  | *** |
|  |  |  | 8 (+/-9) | **** |

**Table S3**

**Hu-BLT mice (No injection + no tooth extraction)**

| **BM**  **(n=4)** | **Spleen**  **(n=5)** | **PBMCs**  **(n=3)** | **P values** |
| --- | --- | --- | --- |
| 32 +/- 8 | 258 +/-54 |  | *** |
| 32 +/- 8 |  | 1696 +/- 123 | **** |

**Hu-BLT (NACL+ tooth extraction) week 2**

| **BM**  **(n=4)** | **Spleen**  **(n=4)** | **PBMCs**  **(n=4)** | **Gingiva**  **(n=2)** | **Pancreas**  **(n=3)** | **p values** |
| --- | --- | --- | --- | --- | --- |
| 51 (+/-12) | 254 (+/-40) |  |  |  | **** |
| 51 (+/-12) |  | 812 (+/-64) |  |  | **** |
| 51 (+/-12) |  |  | 43 (+/-7) |  | ns |
| 51 (+/-12) |  |  |  | 31 (+/-5) | * |

**Hu-BLT (ZOL + tooth extraction) week 2**

| **BM**  **(n=4)** | **Spleen**  **(n=4)** | **PBMCs**  **(n=4)** | **Gingiva**  **(n=2)** | **Pancreas**  **(n=3)** | **p values** |
| --- | --- | --- | --- | --- | --- |
| 144 (+/-48) | 786 (+/-69) |  |  |  | *** |
| 144 (+/-48) |  | 1702 (+/-184) |  |  | **** |
| 144 (+/-48) |  |  | 14 (+/4) |  | **** |
| 144 (+/-48) |  |  |  | 40 (+/-3) | *** |

**Hu-BLT (NACL+ tooth extraction) week 4**

| **BM**  **(n=4)** | **Spleen**  **(n=4)** | **PBMCs**  **(n=4)** | **Gingiva**  **(n=2)** | **Pancreas**  **(n=3)** | **p values** |
| --- | --- | --- | --- | --- | --- |
| 78 (+/-33) | 388 (+/-26) |  |  |  | **** |
| 78 (+/-33) |  | 1029 (+/-60) |  |  | **** |
| 78 (+/-33) |  |  | 97 (+/-23) |  | ns |
| 78 (+/-33) |  |  |  | 36 (+/-4) | ** |

**Hu-BLT (ZOL + tooth extraction) week 4**

| **BM**  **(n=4)** | **Spleen**  **(n=4)** | **PBMCs**  **(n=4)** | **Gingiva**  **(n=2)** | **Pancreas**  **(n=3)** | **p values** |
| --- | --- | --- | --- | --- | --- |
| 213 (+/-128) | 849 (+/-112) |  |  |  | *** |
| 213 (+/-128) |  | 1987 (+/-170) |  |  | **** |
| 213 (+/-128) |  |  | 51 (+/-9) |  | *** |
| 213 (+/-128) |  |  |  | 48 (+/-3) | **** |

**Table S4**

| **Hu-BLT week 2 (n=4)** | **PBMCs** | | **BM** | | **Spleen** | | **Pancreas** | |
| --- | --- | --- | --- | --- | --- | --- | --- | --- |
|  | **NACL** | **ZOL** | **NACL** | **ZOL** | **NACL** | **ZOL** | **NACL** | **ZOL** |
| **% CD16+CD56+** | **13** | **7.1** | **2.3** | **3** | **3.3** | **4.1** | **2.3** | **2.3** |
| **% CD3+** | **51** | **68.3** | **16.5** | **9.1** | **28.3** | **49.3** | **41** | **58** |

**Figure legends**

**Figure S1: IFN-γ secretion by PBMCs, BM, spleen, and gingiva of NACL and ZOL-injected Hu-BLT mice.**

Hu-BLT mice were administered with either 0.9% NACL or ZOL (500 µg/kg) via IV as described in Materials and Methods section. Four weeks after injections, mice were euthanized and tissues were harvested to obtain single cell suspension. BM cells, splenocytes, PBMCs and gingiva cells of hu-BLT mice were cultured (2 × 10^6^ cells/2ml) without or with IL-2 (1000 U/ml) for three days, after which the supernatants were harvested and the levels of IFN-γ was determined using single ELISA. (n=2)

**Figure S2: Immune cell composition of PBMCs, BM and spleen in NACL and ZOL-injected Hu-BLT mice.**

Hu-BLT mice were administered with either 0.9% NACL or ZOL (500 µg/kg) via IV as described in Materials and Methods section. Four weeks after injections, mice were euthanized and tissues were harvested to obtain single cell suspension. Immune cell composition was determined in BM (n=2), splenocytes (n=2), and PBMCs (n=2) of hu-BLT mice using flow cytometric analysis.

**Figure S3: Immune cell percentages in oral gingival cells of NACL or ZOL-injected and tooth extracted Hu-BLT mice.**

Hu-BLT mice were administered with either 0.9% NACL or ZOL (500 µg/kg) via IV followed by maxillary left first molar extraction as described in Materials and Methods section. Four weeks after injections, mice were euthanized and oral gingival tissues were harvested to obtain single cell suspension. Surface expression of CD45+CD3+, CD45+CD16+CD56+, and CD45+CD3+gdT+ in oral gingival cells were determined using antibody staining with flow cytometric analysis as described in the Materials and Methods.

**Figure S4: IFN-γ secretion by PBMCs, BM, spleen and pancreas of NACL or ZOL-injected and tooth-extracted Hu-BLT mice**

Hu-BLT mice were administered with either 0.9% NACL or ZOL (500 µg/kg) via IV followed by maxillary left first molar extraction as described in Materials and Methods section. Two or four weeks after tooth extraction, mice were euthanized and tissues were harvested to obtain single cell suspension. Cells were cultured (2 × 10^6^ cells/2ml) with IL-2 (1000 U/ml) for three days, after which the supernatants were harvested and the levels of IFN-γ was determined using single ELISA. *****(p value<0.0001), ***(p value <0.001), **(p value 0.001-0.01), *(p value 0.01-0.05).*

**Figure S5: Cytokine secretion by PBMCs, BM, and spleen of NACL and ZOL-injected Hu-BLT mice.**

Hu-BLT mice were administered with either 0.9% NACL or ZOL (500 µg/kg) via IV as described in Materials and Methods section. Two weeks **(A)** and four weeks **(B)** after injections, mice were euthanized and tissues were harvested to obtain single cell suspension. PBMCs, BM cells, and splenocytes of hu-BLT mice were cultured (2 × 10^6^ cells/2ml) with IL-2 (1000 U/ml) for three days, after which the supernatants were harvested and the levels of IFN-γ, TNF-α, IL-6 were determined using multiplex assay. *****(p value<0.0001), ***(p value <0.001), **(p value 0.001-0.01), *(p value 0.01-0.05).*

**Figure S6: Immune cell percentages in PBMCs, BM, spleen and pancreas of NACL or ZOL-injected and tooth-extracted Hu-BLT mice**

Hu-BLT mice were administered with either 0.9% NACL or ZOL (500 µg/kg) via IV followed by maxillary left first molar extraction as described in Materials and Methods section. Two or four weeks after tooth extraction, mice were euthanized and tissues were harvested to obtain single cell suspension. Immune cell composition was determined in PBMCs (n=4), BM (n=4), splenocytes (n=4), and pancreas (n=3) of hu-BLT mice using flow cytometric analysis. *****(p value<0.0001), ***(p value <0.001), **(p value 0.001-0.01), *(p value 0.01-0.05).*

**Figure S7: Immune cell composition of PBMCs, BM and spleen in NACL and denosumab-injected hu-BLT mice.**

Hu-BLT mice were administered with either 0.9% NACL or denosumab (????) via IV as described in Materials and Methods section. Four weeks after injections, mice were euthanized and tissues were harvested to obtain single cell suspension. Immune cell composition was determined in BM (n=2), splenocytes (n=2), and PBMCs (n=2) of hu-BLT mice using flow cytometric analysis.

**Fig. S1**

**Fig. S2**

**Fig. S3**


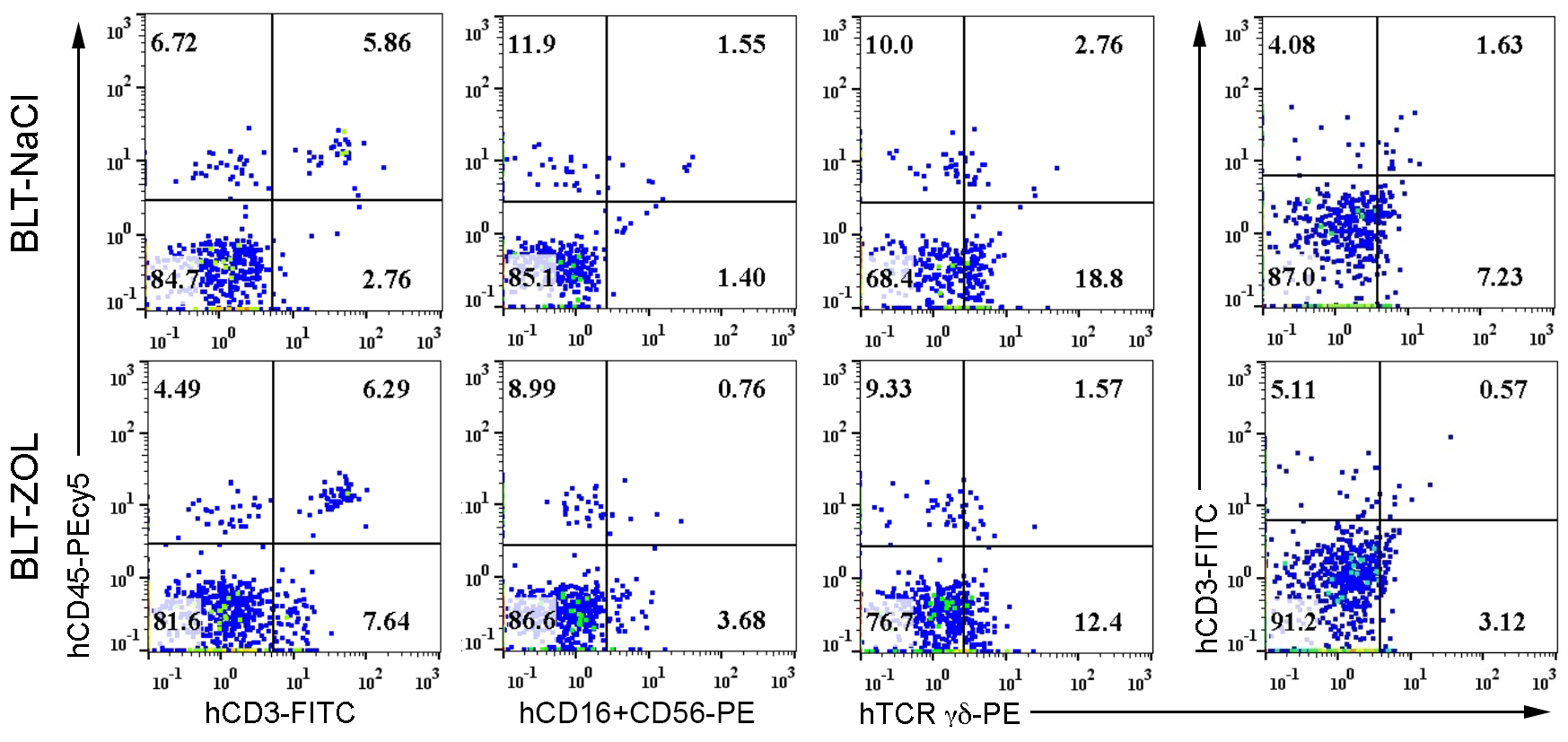


**Fig. S4**

**
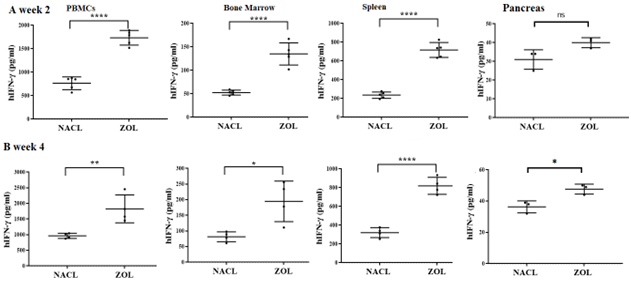
**

**Fig. S5**

**
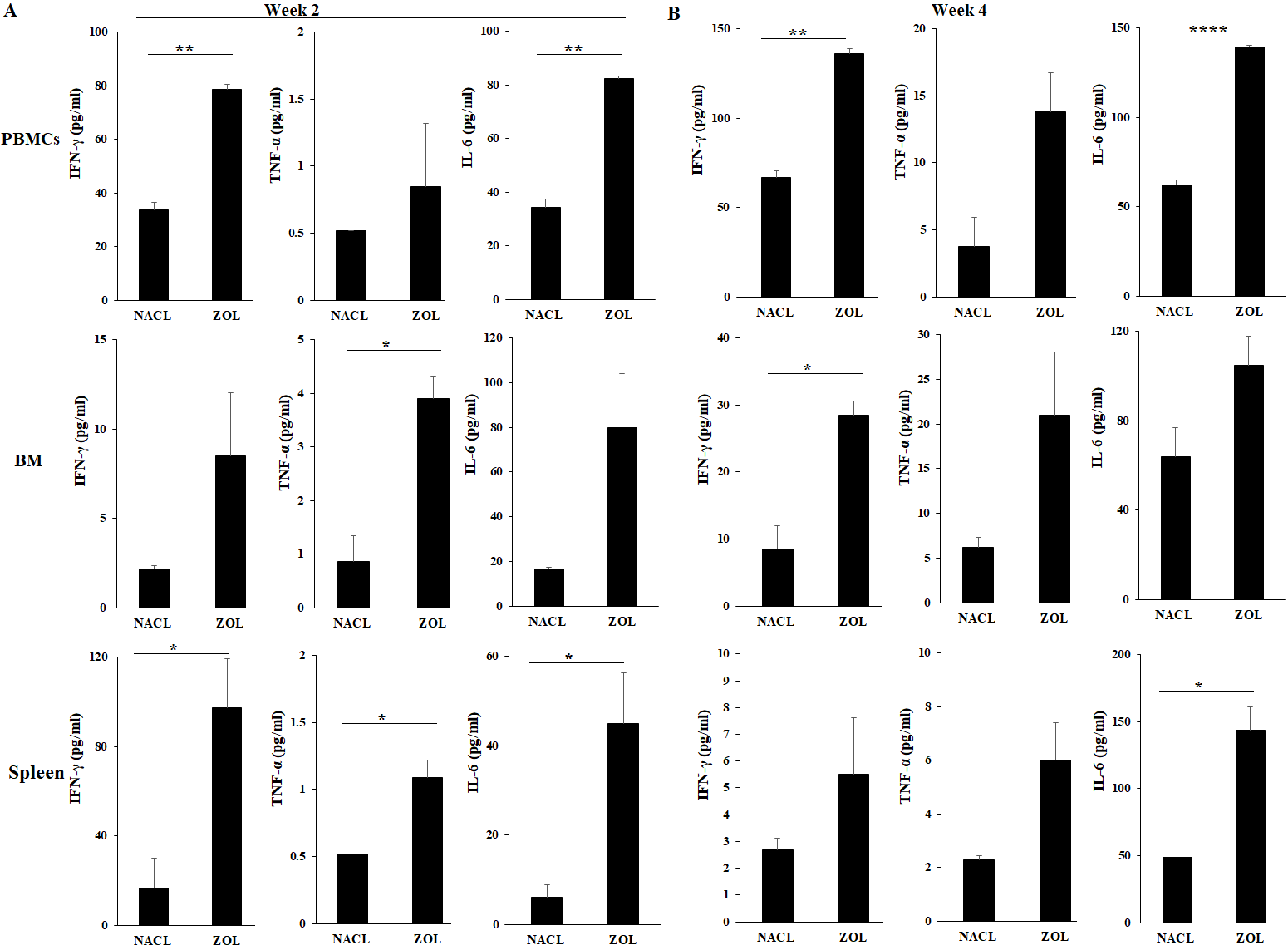
**

**Fig. S6**

**Fig. S7**
